# Fast and Slow Effects of Medial Olivocochlear Efferent Activity in Humans

**DOI:** 10.1371/journal.pone.0018725

**Published:** 2011-04-08

**Authors:** Wei Zhao, Sumitrajit Dhar

**Affiliations:** 1 Roxelyn and Richard Pepper Department of Communication Sciences and Disorders, Northwestern University, Evanston, Illinois, United States of America; 2 Interdepartmental Neuroscience Program, Northwestern University, Evanston, Illinois, United States of America; 3 Hugh Knowles Center, Northwestern University, Evanston, Illinois, United States of America; Tokyo Medical and Dental University, Japan

## Abstract

**Background:**

The medial olivocochlear (MOC) pathway modulates basilar membrane motion and auditory nerve activity on both a fast (10–100 ms) and a slow (10–100 s) time scale in guinea pigs. The slow MOC modulation of cochlear activity is postulated to aide in protection against acoustic trauma. However in humans, the existence and functional roles of slow MOC effects remain unexplored.

**Methodology/Principal Findings:**

By employing contralateral noise at moderate to high levels (68 and 83 dB SPL) as an MOC reflex elicitor, and spontaneous otoacoustic emissions (SOAEs) as a non-invasive probe of the cochlea, we demonstrated MOC modulation of human cochlear output both on a fast and a slow time scale, analogous to the fast and slow MOC efferent effects observed on basilar membrane vibration and auditory nerve activity in guinea pigs. The magnitude of slow effects was minimal compared with that of fast effects. Consistent with basilar membrane and auditory nerve activity data, SOAE level was reduced by both fast and slow MOC effects, whereas SOAE frequency was elevated by fast and reduced by slow MOC effects. The magnitudes of fast and slow effects on SOAE level were positively correlated.

**Conclusions/Significance:**

Contralateral noise up to 83 dB SPL elicited minimal yet significant changes in both SOAE level and frequency on a slow time scale, consistent with a high threshold or small magnitude of slow MOC effects in humans.

## Introduction

The medial olivocochlear (MOC) pathway, a part of the auditory efferent system, has gained increasing attention in recent years. Its postulated functional roles include protection against acoustic trauma and facilitation of transient-sound perception in a noisy background [1], [2]. Projecting from the medial region of the superior olivary complex, the MOC fibers innervate cochlear outer hair cells (OHCs) via cholinergic synapses. The MOC modulation of the cochlea operates on two time scales, three orders of magnitude apart. Activation of the MOC fibers opens the postsynaptic acetylcholine-gated α9/α10 channels [3], [4], leading to a calcium influx into OHCs [5], [6], [7]. Subsequently, a potassium outflow through calcium-activated potassium channels hyperpolarizes OHCs and decreases the gain of the cochlear amplifier. Hence on a fast time scale (10–100 ms), basilar membrane motion is inhibited and auditory nerve activity reduced. The MOC activity on a slow time scale (10–100 s), although not extensively studied, has been linked to the slow calcium release from intracellular stores and the decrease in OHC's axial stiffness [8], [9], [10]. Both fast and slow MOC effects reduce auditory nerve activity and basilar membrane vibration amplitude in guinea pigs [8], [11], [12]. The fast and slow MOC effects on the phase of basilar membrane displacement, however, are in opposite directions: fast effects causing phase leads and slow effects, phase lags [8]. Furthermore, slow effects peak at a higher frequency than fast MOC effects [12].

MOC effects on the auditory periphery can also be observed using a non-invasive, albeit indirect, method via otoacoustic emissions (OAEs). Spontaneous OAEs (SOAEs) are sounds generated in the cochlea without external stimulation [13]. Their generation is modeled either by standing-wave resonance in the cochlea [13], or by active autonomous oscillation of the stereocilia [18]. Elicited acoustically, the MOC reflex reduces SOAE level and increases its frequency [19], [20], [21], [22], [23] on a time scale consistent with the fast MOC effects observed on basilar membrane mechanics. However, studies in humans have yet to distinguish slow from fast MOC effects.

The present study demonstrates the presence of slow MOC effects on SOAEs in humans. The magnitude of slow MOC effects was miniscule in comparison to that of fast effects.

## Methods

### Subjects

Thirteen human subjects (ten female and three male), between the ages of 20 and 30 years with normal hearing sensitivity (20 dB HL or better at octave frequencies between 250–8000 Hz, measured with an Interacoustics Audio Traveller AA220) in both ears were recruited for this study. Experiments were conducted on one ear per subject (ten right and three left ears). All subjects selected for the experiments had at least one SOAE 10 dB above the noise floor. All procedures were approved by the Northwestern University Institutional Review Board (Northwestern IRB #4X - Panel E, registration number IRB00000736). Written, informed consent was obtained from each subject. Measurements were conducted in a sound-treated audiological test booth.

### Signal generation and recording

Digital stimuli (sampling rate 44100 Hz, 24 bit) were generated by a Macintosh computer and converted to analog signals by a MOTU 828 MKII input/output device. Stimuli were presented to subjects via MB Quart 13.01 HX transducers coupled to the ear canal with an Etymotic Research ER10B+ probe. OAE signals were acquired in subjects' ear canals using the ER10B+ microphone, amplified by a pre-amplifier (+20 dB), digitized by the MOTU and stored on disk for processing offline.

### Fast and slow MOC effects on SOAEs

**Figure 1:**
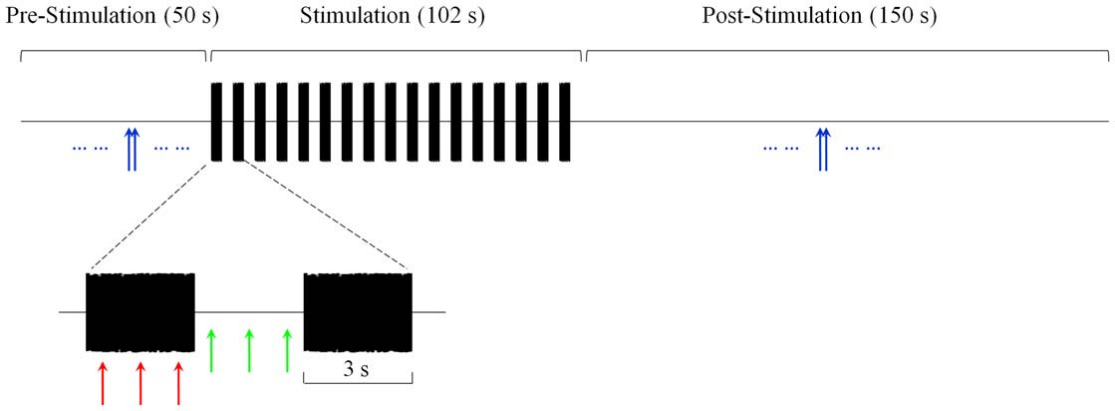
Schematic of the experimental paradigm. Each SOAE was monitored for 302 s. Baseline SOAE level and frequency were established in the pre-stimulation window. Contralateral broadband noise pulses (3-s long, 68 dB SPL) were presented in the 102-s stimulation window with 3-s inter-pulse intervals. Blue arrows represent SOAE estimates in the pre- and post-stimulation windows, red arrows during noise pulses, and green arrows during inter-pulse intervals. This color convention is consistent throughout the paper. Each arrow represents an estimate of SOAE level or frequency averaged over a 1-s window.

MOC efferents were activated by a contralateral broadband noise (100–10000 Hz, 68 dB SPL, 5 ms rise/fall time). A higher-level (83 dB SPL) contralateral noise was applied to three subjects in a subset of the experiments. Each run consisted of a pre-stimulation window (50 s), a stimulation window (102 s) and a post-stimulation window (150 s) (Figure 1). As an acoustical approximation of the stimulation paradigm employed by Sridhar et al. (1995) and Cooper and Guinan (2003) [8], [12], 3-s noise bursts were presented in the contralateral ear interleaved with 3-s silent intervals within the stimulation window. A total of six runs were recorded and a zero-padded fast Fourier transform (FFT) was performed for each 1-s window to generate SOAE spectra with a frequency resolution of 0.5 Hz. The noise floor of each 1-s window was taken as the median spectral level in a 100-Hz range surrounding the target SOAE. SOAE data were rejected off-line if SOAE level in the local time window was less than two standard deviations above the average noise floor for the entire recording.

### Middle-ear reflex test

The middle-ear muscle (MEM) and MOC reflexes share the same afferent pathway and are elicited by external sounds of similar levels. Hence, it is critical to ensure the MEM reflex remains dormant while the MOC reflex is elicited. Two experimental methods, the group delay method [24] and the suppression method [25], were implemented in all subjects to monitor the MEM reflex. Both methods utilize stimulus frequency OAEs (SFOAEs), which are low-level acoustic signals evoked by a single-frequency tone and measured in the ear canal [26]. SFOAEs can be extracted by nonlinear compression, two-tone suppression, or spectral smoothing [27]. Both suppression and compression techniques were used in our MEM contraction test to extract SFOAEs evoked by a 40 dB SPL probe tone.

In the group delay method, the vector difference between the total ear canal pressure at the probe frequency with and without contralateral noise was denoted ΔP. The group delay of ΔP was computed as the negative of its phase slope. Around 1500 Hz, a ΔP group delay near 10 ms indicates the dominance of the MOC reflex over the MEM reflex [24], [28]. The group delay of ΔP was measured using both a sweeping probe tone paradigm and a discrete probe tone paradigm (Figure S1), as described below. In the sweeping probe tone paradigm, a probe was swept from 800 to 2400 Hz in 12 s. The following triplet was repeated eight times: a 40 dB SPL probe in the absence of contralateral noise, followed by a 60 dB SPL probe in the absence of contralateral noise, and finally a 40 dB SPL probe paired with contralateral noise. Averaged data were passed through an adaptive least-squares fit filter to estimate the level and phase of the total ear canal pressure at the probe frequency [29]. Baseline SFOAE was calculated using the presumed nonlinear compressive growth of SFOAEs [26]. ΔP was also calculated as described above. In the discrete probe paradigm, a discrete tone at 40 dB SPL was presented over an 80-Hz range in 20-Hz steps. The probe tone lasted 5 s, during which time a 68 dB SPL contralateral noise was presented from 0.5 to 3 s. A total of eight runs were performed for each probe frequency and averaged data were passed through an adaptive least-squares-fit filter to obtain probe level and phase.

In the suppression method, SFOAEs were evoked by a 40 dB SPL probe tone at three frequencies (602 Hz, ∼1000 Hz, ∼2000 Hz) in each subject. The probe frequencies around 1000 Hz and 2000 Hz were chosen to be at a local peak of the ΔP versus probe frequency function obtained using the sweeping tone paradigm described earlier. Each recording block in this paradigm lasted 12.5 s (Figure S2A) and was divided into five segments. Four such blocks were averaged to obtain usable signal-to-noise ratios. A probe tone (P) was presented during the entire 12.5 s; a 65 dB SPL suppressor tone (S), 0.1 octaves below the probe, was presented between 2.5 and 7.5 s; and a contralateral broadband noise (C) was presented between 5 and 10.5 s. Thus, the resulting five segments were: probe alone (P), probe plus suppressor (P+S), probe plus suppressor and contralateral noise (P+S+C), probe plus contralateral noise (P+C), and probe alone again (P′). The total ear canal pressure at the probe frequency was measured for each of the five segments. The vector difference between P and P+S yields baseline SFOAE , between P+S and P+S+C yields the pressure change due to potential MEM contraction (blue arrow, Figure S2B), between P and P+C yields ΔP (red arrow, Figure S2B), and between P and P′ is a measure of drift in our measurements (green arrow, Figure S2B). The SFOAE is arguably largely suppressed in the P+S window. Therefore, any differences between the complex pressures in the ear canal measured in the P+S and P+S+C segments can be attributed to the MEM reflex [25]. When the magnitude of ΔP is substantially larger than that of, and therefore cannot be explained by either MEM reflex-induced pressure change or probe drift, ΔP is considered to be dominated by the MOC reflex. For two out of three probe frequencies (∼1000 Hz and ∼2000 Hz), the exemplar subject in Figure S2C displayed a ΔP (red symbols) that was substantially larger than both pressure change by MEM reflex (blue symbols) and probe drift (green symbols), indicating the dominance of the MOC reflex.

Using the above two methods, we ensured the absence of MEM contraction under a 68 dB SPL contralateral noise.

## Results

Effects of contralateral noise on forty-four SOAEs from thirteen subjects, between 871 and 14864 Hz in frequency, and −7 and 18 dB SPL in level, were examined on a fast time scale (evaluated during 3-s bursts of contralateral noise) and on a slow time scale (measured in a 30-s window after noise stimulation). SOAE levels were reduced on both the fast and the slow time scale. SOAE frequencies were elevated on the fast time scale but reduced on the slow time scale. The magnitude of fast effects was significantly greater than that of slow effects.

### Demonstration of MOC-induced changes in SOAEs

**Figure 2:**
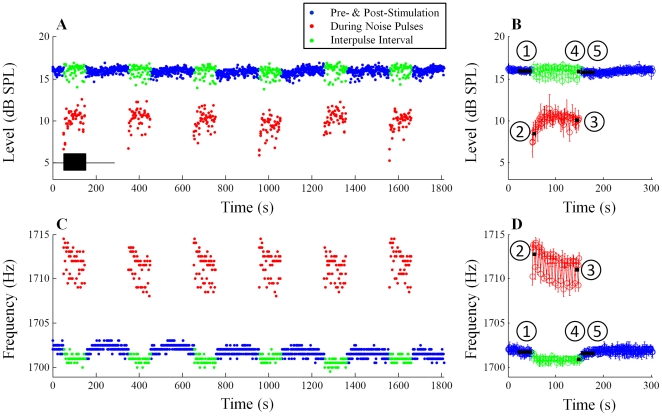
Example of MOC effects on one SOAE (subject WTPF01). SOAE level over six consecutive repetitions of the stimulation paradigm (A), and averaged level over six repetitions (C) are shown in the top row (blue: pre- and post-stimulation windows; red: during noise pulses; green: inter-pulse intervals). The black rectangular box in Panel A represent the noise pulses which are shown only for the first experimental run but were repeated six times. Baseline (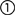) is defined as the median SOAE level/frequency in the 30-s window before noise onset. Similarly, SOAE levels/frequencies during the first two noise pulses (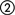), during the last two noise pulses (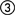), during the last two inter-pulse intervals (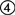) and in the 30-s window after noise offset (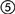) were quantified. Differences from the baseline were defined as *fast change* (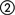-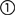), *adaptation change* (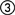-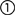), *buildup change* (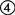-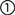) and *slow change* (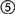-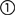). Error bars represent one standard deviation.

An example of MOC-induced changes in SOAE level and frequency is presented in Figure 2 (SOAE at 1702 Hz/16 dB SPL, subject WTPF01). Six consecutive trials of the 302-s noise stimulation paradigm (represented by the black rectangular box in Figure 2A), totaling 1812 s, were recorded from each subject. SOAE level and frequency throughout the entire 1812 s are shown in Figure 2A and 2C, respectively. SOAE level and frequency averaged over six runs are presented in Figure 2B and 2D. Blue symbols depict estimates of SOAE level (Figure 2A, 2B) and frequency (Figure 2C, 2D) in the pre- and post-stimulation windows. Estimates of SOAE level and frequency during and between noise pulses are presented using red and green symbols, respectively.

Baseline values of SOAE level and frequency were established by averaging these values over the 30-s window before noise onset (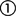 in Figure 2B, 2D). Noise pulses suppressed SOAE level (red symbols, Figure 2A, 2B) and elevated SOAE frequency (red symbols, Figure 2C, 2D). Differences between the estimates of SOAE level and frequency during the first two noise pulses (0–3 & 6–9 s of the stimulation window, 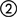 in Figure 2B, 2D) and the baseline were defined as *fast changes* (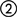-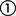). *Fast changes* represent fast MOC effects as the measurement epoch was limited to the first few seconds of the stimulation window, thereby eliminating the influence of slow MOC effects. As evident in Figure 2B and 2D, changes in both SOAE level and frequency gradually adapted over tens of seconds during noise pulses. Differences between SOAE estimates during the last two noise pulses (90–93 & 96–99 s of the stimulation window, 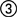 in Figure 2B, 2D) and the baseline were defined as *adaptation changes* (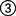-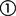). This metric reflects the mixed effects of adapted fast and slow MOC effects. Residual SOAE changes in the inter-pulse intervals and after noise offset were much smaller than changes during noise pulses. Differences between estimates of SOAE level and frequency during the last two inter-pulse intervals (93–96 & 99–102 s of the stimulation window, 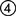 in Figure 2B, 2D) and the baseline were defined as *buildup changes* (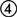-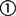). *Buildup changes* are free of fast MOC effects, but manifest a mixture of slow MOC effects and a post-noise overshoot [23]. Differences between averaged SOAE estimates in the first 30 s of the post-stimulation window (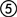 in Figure 2B, 2D) and the baseline were defined as *slow changes* (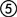-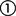). *Slow changes* are a pure representation of slow MOC effects, as both fast MOC effects and the post-noise overshoot dissipate in the first few seconds of the post-stimulation window. Both *buildup* and *slow changes* were smaller in magnitude than *fast* and *adaptation changes*. Group data are presented below.

### Group Results

**Figure 3:**
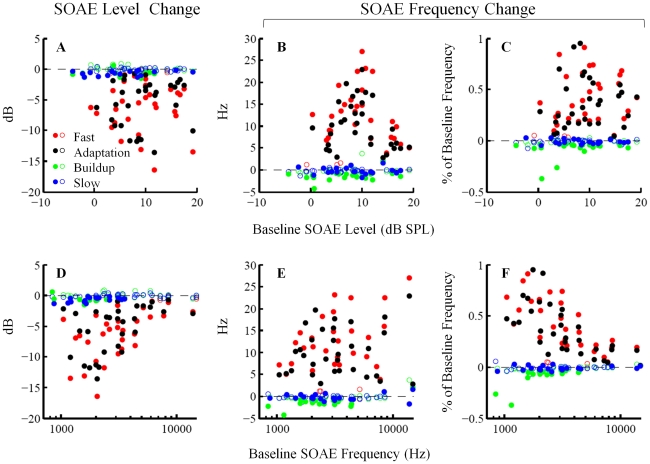
Scatter plot of MOC-induced changes in SOAE level (left column) and frequency (middle and right columns) as functions of baseline SOAE level (upper row) and frequency (lower row). Filled and open symbols represent statistically significant (*t*-test, *p*<0.01) and non-significant (*t*-test, *p*≥0.01) changes from baseline, respectively. *Fast* and *adaptation changes* are larger in magnitude than *buildup* and *slow changes*.

**Figure 4:**
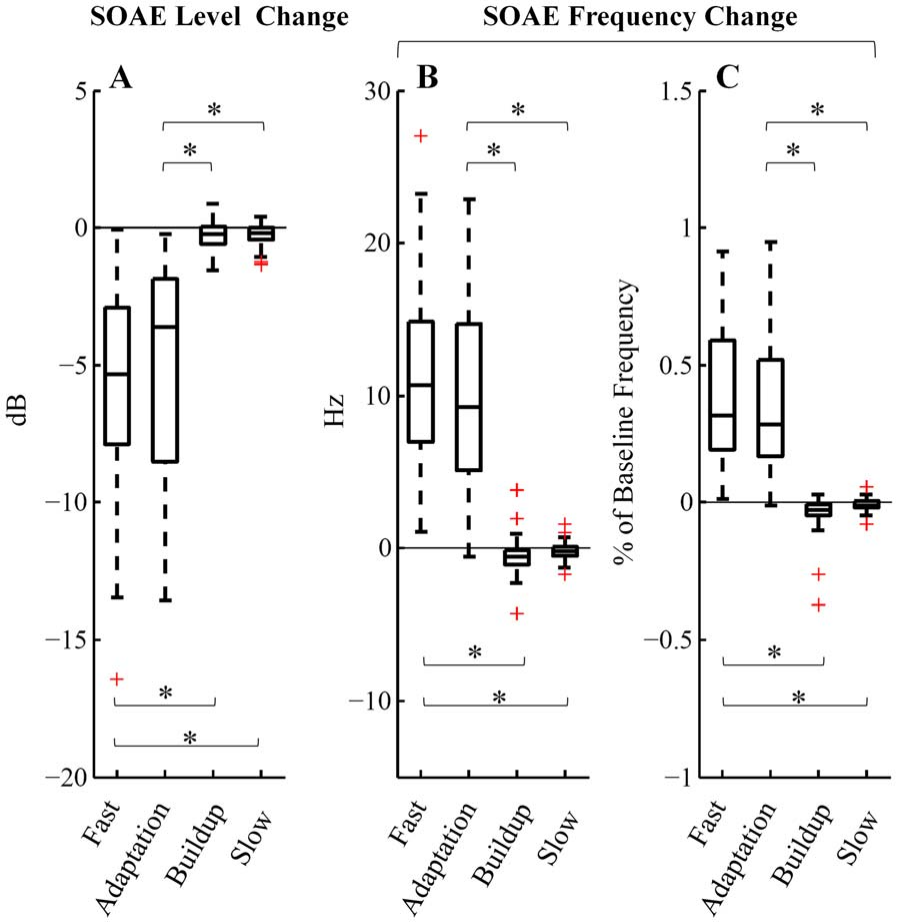
Comparison of magnitudes of *fast*, *adaptation*, *buildup* and *slow changes* (N = 32, 33, 44, 44, respectively) in SOAE level (A) and frequency (B, C). The results of a paired Wilcoxon signed-rank test demonstrated that *slow* and *buildup changes* were significantly smaller than *fast* and *adaptation changes* (*: *p*<1e-6). The central line on each box is the median value. The top and bottom edge lines represent 25^th^ and 75^th^ percentiles, respectively. Whiskers cover all data points within 1.5 interquartile range from the top and bottom edge lines. Red crosses mark outliers beyond the whiskers.

*Fast*, *adaptation*, *buildup* and *slow changes* were quantified for individual SOAEs (N = 44) and are presented in Figure 3. SOAE level changes in dB (left column, Figure 3), SOAE frequency changes in Hz (middle column, Figure 3) and SOAE frequency change as percentage of baseline frequency (right column, Figure 3) are plotted as functions of baseline SOAE level (upper row, Figure 3) and frequency (lower row, Figure 3). Filled and open symbols represent statistically significant (*t*-test, *p*<0.01) and non-significant (*t*-test, *p*≥0.01) changes from baseline, respectively. This convention to distinguish significant from non-significant changes is preserved throughout the paper. The grand averages for *fast change* (N = 32), *adaptation change* (N = 33), *buildup change* (N = 44) and *slow change* (N = 44) are presented in Figure 4. The analysis exclusion criterion (excluding SOAEs less than two standard deviation above the local noise floor) accounts for the discrepancy between the overall sample size (N = 44) and the sample sizes of *fast change* (N = 32) and *adaptation change* (N = 33).

*Fast* and *adaptation changes* manifested as decrease in SOAE level and elevation in SOAE frequency (Wilcoxon signed-rank test, *p<*1e-6) (Figures 3 and 4). The median *fast changes* were −5.3 dB in level and 10.7 Hz in frequency (0.32% of baseline frequency). The median *adaptation changes* were −3.6 dB in level and 9.3 Hz in frequency (0.28% of baseline frequency). Note that we implemented an analysis exclusion criterion, which rejects SOAEs less than two standard deviations above the local noise floor. Since low-level SOAEs are more likely to be suppressed into or near the noise floor and thus be excluded from analyses, *fast* and *adaptation changes* as functions of baseline SOAE level (upper row, Figure 3) could have been tainted by this exclusion criterion, which probably accounts for the trend that SOAEs below 5 dB SPL had less *fast* and *adaptation changes* in level than larger SOAEs (Figure 3A). No prominent feature was observed in the *fast* and *adaptation changes* versus baseline SOAE level functions. *Fast* and *adaptation changes* as functions of baseline SOAE frequency (lower row, Figure 3) however demonstrated clear patterns. *Fast* and *adaptation changes* in both level (in dB) and frequency (as percentage of baseline frequency) were greater for low- to mid-frequency SOAEs than for high-frequency SOAEs (Figure 3D and 3F).

*Buildup* and *slow changes* manifested as reductions in both SOAE level and frequency (Wilcoxon signed-rank test, *p<*0.01) (Figures 3 and 4). The median *buildup changes* were −0.2 dB in level and −0.6 Hz in frequency (−0.02% of baseline frequency). The median *slow changes* were −0.2 dB in level and −0.2 Hz in frequency (−0.01% of baseline frequency). No dependence of *buildup* or *slow changes* on either baseline level or frequency could be easily identified.

The paired Wilcoxon signed-rank test was performed to compare the four types of changes. Different ears emitted different numbers of SOAEs at diverse frequencies and levels, and each SOAE was treated as an independent observation. *Fast* and *adaptation changes* were significantly larger in magnitude than *buildup* and *slow changes* (Paired Wilcoxon signed-rank test, *p*<1e-6) (Figure 4).

**Figure 5:**
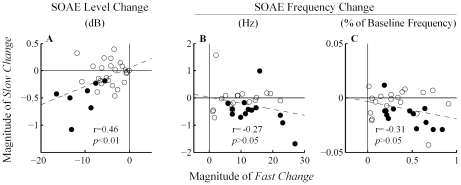
Correlation between MOC-induced *fast* and *slow changes* in SOAE level (A) and frequency (B, C). Filled symbols indicate that both *fast* and *slow changes* were statistically significant (*t*-test, *p*<0.01), whereas open symbols indicate otherwise. A positive correlation for SOAE level was observed. Dashed lines represent best linear fits to all of the data points.

To demonstrate the relationship between *fast* and *slow changes*, *fast changes* are plotted against *slow changes* (N = 32) (Figure 5). Filled symbols indicate that both *fast* and *slow changes* were statistically significant (*t*-test, *p*<0.01), whereas open symbols indicate otherwise. A weak positive correlation (r = 0.46, *p*<0.01) was observed between *fast* and *slow changes* in SOAE level (both filled and open symbols included), consistent with a sequential relationship in a series of physiological events. Correlation between *fast* and *slow changes* in SOAE frequency was not statistically significant (*p*>0.05).

### Effect of contralateral noise level on slow changes

The contralateral noise level of 68 dB SPL was selected for our experiments in order to avoid elicitation of the MEM reflex. This mild noise level may have led to insufficient activation of the MOC bundle, resulting in the relatively small *slow changes* in SOAEs (Figures 3 and 4). To study the influence of contralateral noise level on the magnitude of *slow changes*, we repeated the experiments in three subjects on a different day with a higher noise level of 83 dB SPL. The *slow changes* were evaluated over a 30-s post-stimulation window while the potential MEM reflex has a time constant in the range of hundreds of milliseconds [30]. Given these differences in the time courses between slow MOC effects and the MEM reflex, MEM contraction, if elicited by the 83 dB SPL noise, arguably had little influence on the *slow changes*.

**Figure 6:**
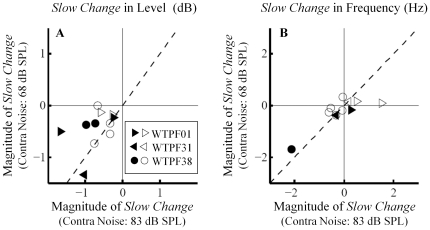
Comparison of *slow changes* elicited by two contralateral noise levels, 68 and 83 dB SPL, in SOAE level (A) and frequency (B) (subjects WTPF01, WPTF31 and WTPF38). Filled symbols indicate *slow changes* elicited by both noise levels were statistically significant (*t*-test, *p*<0.01), whereas open symbols indicate otherwise. No prominent elicitor-level effect on *slow changes* was observed.

SOAEs that were present on both days were examined in detail (N = 11). Increasing noise level from 68 to 83 dB SPL did not substantially enhance the magnitude of *slow changes* (Figure 6). Filled symbols indicate *slow changes* elicited by both noise levels were statistically significant (*t*-test, *p*<0.01), whereas open symbols indicate otherwise. Data points cluster along the diagonal, suggesting no increase in the magnitude of slow effects as the noise level increased from 68 to 83 dB SPL. The reader is reminded that the changes should be evaluated in their absolute values (i.e., a value of −2 dB is a greater change than a value of −1 dB).

## Discussion

The discovery of MOC modulation of cochlear output on a slow time scale (10–100 s) in guinea pigs prompted speculations on its functional roles in protection against acoustic trauma [10], [12], [31]. Here we demonstrate that contralateral noise (68 and 83 dB SPL) elicits miniscule yet significant slow MOC effects on SOAEs in human subjects, consistent with a high threshold or small magnitude of slow MOC effects.

### Interference from MEM contraction

Since the MOC reflex and the MEM reflex share similar temporal characteristics, and can both be elicited by contralateral broadband noise, it is difficult to parse the modulation of OAEs by these two reflexes. In this work, absence of MEM contraction when applying a 68 dB SPL contralateral noise was ensured using two experimental methods, the group delay method and the suppression method. In the group delay method, the group delay of ΔP induced by contralateral noise was around 10 ms in all subjects (Figure S1). In the suppression method, three probe frequencies were applied (602 Hz, ∼1000 Hz and ∼2000 Hz), and ΔP was substantially larger than the pressure change caused by MEM reflex or probe drift for at least two of the three probe frequencies (Figure S2). Nonetheless, substantial probe drift or the minimal magnitude of ΔP prevented unambiguous exclusion of the MEM reflex at some probe frequencies. Overall, these controls made us reasonably confident that our findings were not strongly influenced by MEM contraction. Thus, the slow modulation of SOAEs can be attributed to slow effects of the MOC efferents.

### Fast and slow MOC effects on human SOAEs

Both fast and slow MOC effects led to a reduction of SOAE level in humans (Figure 4A). Consistent with human data, both fast and slow MOC effects in guinea pigs reduce basilar membrane motion and auditory nerve activity [8], [11], [12]. The reduction in all these cochlear measures stems from the MOC-induced attenuation of the gain of the cochlear amplifier.

Fast MOC effects led to an elevation in SOAE frequency whereas slow MOC effects cause a reduction (although small) in SOAE frequency (Figure 4B and 4C). In guinea pigs, fast and slow MOC effects have been shown to produce phase changes of basilar membrane motion in opposite directions, fast effects producing phase leads and slow effects phase lags [8], [32]. Congruity between OAE and basilar membrane data can be achieved under the framework of the global standing wave model of SOAEs [15], where phase leads in basilar membrane vibration predict elevation in SOAE frequency, and phase lags predict decrease in SOAE frequency [23].

The magnitude of fast MOC effects on both SOAE level and frequency (as percentage of baseline frequency) appears to diminish gradually above 6 kHz with increasing frequency (Figure 3D and 3F). This is in agreement with the frequency dependence of fast MOC effects in guinea pigs, which drops off above 10 kHz [12].

### Comparing slow MOC effects between humans and guinea pigs

In guinea pigs, the magnitudes of slow and fast MOC effects are not drastically different. The magnitude of the slow MOC suppression of auditory nerve activity peaks at a higher frequency (∼16 kHz) compared to that of fast MOC suppression (6–10 kHz), and the peak magnitude of slow effects is three to four times smaller than that of fast effects [11], [12]. Fast and slow MOC effects on basilar membrane vibration are also comparable in size [8].

In contrast to data obtained from guinea pigs, slow MOC effects on human SOAEs are strikingly small compared to fast MOC effects (Figure 4). The median slow effects were −0.2 dB (level change) and −0.2 Hz (frequency change), whereas the median fast effects were −5.3 dB and 10.7 Hz.

Although we highlight the miniscule magnitude of slow MOC effects in our data, we did not fully account for their frequency dependence. While slow effects peak at high frequencies (∼16 kHz) in guinea pigs [12], the propensity of measurable SOAEs to cluster in the low frequency range (1–4 kHz) in humans limited our scope. However, it bears mention that *slow changes* in SOAE level or frequency were minimal for SOAEs above ∼6 kHz (Figure 3B, 3D). The lack of pronounced slow effects across all frequencies evaluated in humans may be due to differences in species and in experimental design (e.g., stimulus, stimulation paradigm, measures, etc.).

Another critical difference between our experiments and those in guinea pigs is the intensity of MOC efferent stimulation. The shock rate of 150/s used in the continuous paradigm of Sridhar et al. (1995) is roughly equivalent to acoustic stimulation over 90 dB SPL [12]. Hence, our 68 dB SPL contralateral noise is much weaker an elicitor of efferent activity than electrical shocks delivered to guinea pigs. To partially address this difference in elicitor intensity, we examined three subjects with a higher-level contralateral noise at 83 dB SPL but observed no substantial increase in the magnitude of slow effects (Figure 6).

Arguably, the slow effects in this study are qualitatively similar to those in guinea pigs. With a stimulation paradigm that was an acoustical approximation of that employed by Sridhar et al. (1995) and Cooper and Guinan (2003), we observed a positive correlation between *fast* and *slow changes* in SOAE level (Figure 5A), consistent with the positive correlation in guinea pigs between fast and slow effects as stimulus level increases [12]. In contrast, Larsen and Liberman (2009) applied lengthened continuous noise stimulation (5-min long) in guinea pigs and reported a negative correlation between ‘onset’ suppression and ‘buildup’ suppression of compound action potential [33]. In mice, electrically shocking the olivocochlear efferents leads to a post-shock enhancement of sound-evoked compound action potential and distortion product OAEs over tens or even hundreds of seconds [34]. This novel enhancement, which is independent of α9 cholinergic receptors, probably does not share common underlying mechanisms as the suppressive slow MOC effects observed in guinea pigs and humans, which reduce the amplitude of cochlear output.

### Conclusions

The auditory efferents are thought to play a protective role against acoustic trauma. This protective role has been associated with slow MOC effects elicited by electrical shocks in guinea pigs [31]. In these experiments, MOC efferents were stimulated by the electrical equivalent of noise over 90 dB SPL. We have shown that contralateral noise up to 83 dB SPL elicited miniscule slow MOC effects in humans. However, our results did not rule out a higher threshold for larger slow effects or rapid growth in the magnitude of slow effects with increasing noise level. In three human subjects, the magnitude of slow effects did not grow dramatically as the level of contralateral noise increased from 68 to 83 dB SPL. It is yet to be seen whether noise over 90 dB SPL produces prominent slow effects. The functional relevance of slow MOC effects remains elusive.

## Supporting Information

Figure S1 **Example of the group delay method for detecting middle-ear muscle (MEM) contraction.** A 40 dB SPL probe tone was either swept across, or presented at discrete frequencies in 20-Hz steps over an 80-Hz range near 1000 Hz. The total ear canal pressure was measured with and without a 68 dB SPL contralateral noise. The magnitude (A) and phase (B) of the vector difference in the total ear canal pressure at the probe frequency between the two conditions, denoted ΔP, was plotted as a function of probe frequency. A group delay of ΔP around 10 ms indicates the dominance of the MOC reflex over the MEM reflex.
(TIF)

Figure S2 **The suppression method for detecting MEM contraction. (A) Illustration of the experimental paradigm.** A 40 dB SPL probe tone, a 65 dB SPL suppressor tone 0.1 octave below the probe tone, and a 68 dB SPL contralateral noise were presented for different durations in a 12.5-s window, segmenting it into five conditions: probe alone (P), probe plus suppressor (P+S), probe plus suppressor and contralateral noise (P+S+C), probe plus contralateral noise (P+C), and finally probe alone again (P′). Computing the vector difference between these conditions yields baseline SFOAE, pressure change induced by the middle-ear muscle reflex (MEMR) (blue arrow), contralateral noise-induced shift ΔP (red arrow) and probe drift (green arrow) (B). Exemplar results from subject WTPF42 are displayed (C). For two SFOAE probe frequencies (∼1000 and ∼2000 Hz), the magnitude of ΔP (red symbols) was substantially larger than that of, and therefore could not be explained by either MEMR-induced pressure change (blue symbols) or probe drift (green symbols). Hence ΔP was considered to be dominated by the MOC reflex. Error bars are one standard error.
(TIF)
