# Supplementary figures and images for: Fast and Slow Effects of Medial Olivocochlear Efferent Activity in Humans
Source: PLoS One. 2011 Apr 8;6(4):e18725. doi: 10.1371/journal.pone.0018725 (PMC3073004; doi:10.1371/journal.pone.0018725)

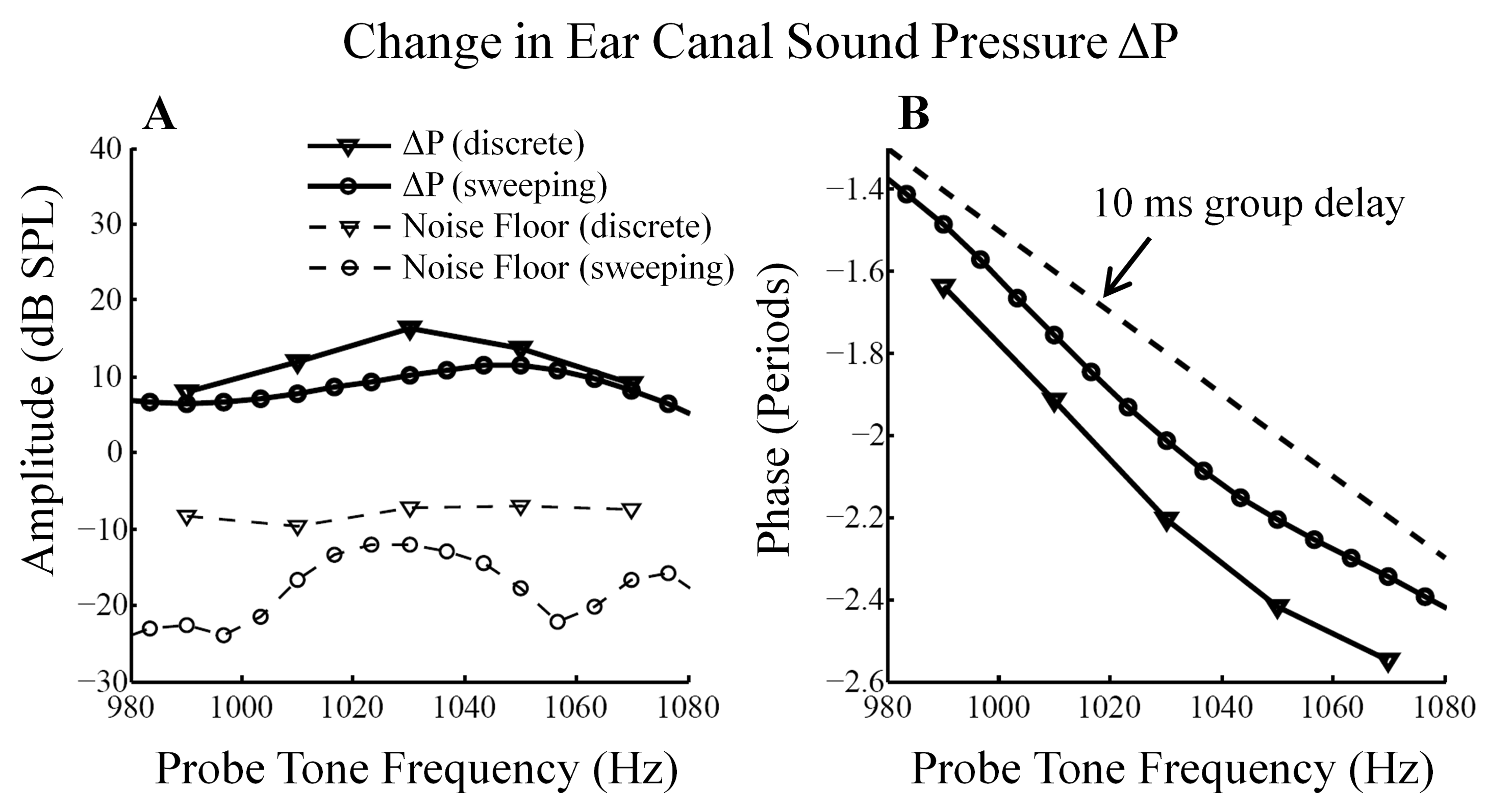

Supplement: Figure S1 — Example of the group delay method for detecting middle-ear muscle (MEM) contraction. A 40 dB SPL probe tone was either swept across, or presented at discrete frequencies in 20-Hz steps over an 80-Hz range near 1000 Hz. The total ear canal pressure was measured with and without a 68 dB SPL contralateral noise. The magnitude (A) and phase (B) of the vector difference in the total ear canal pressure at the probe frequency between the two conditions, denoted ΔP, was plotted as a function of probe frequency. A group delay of ΔP around 10 ms indicates the dominance of the MOC reflex over the MEM reflex. (TIF) [file pone.0018725.s001.tif]

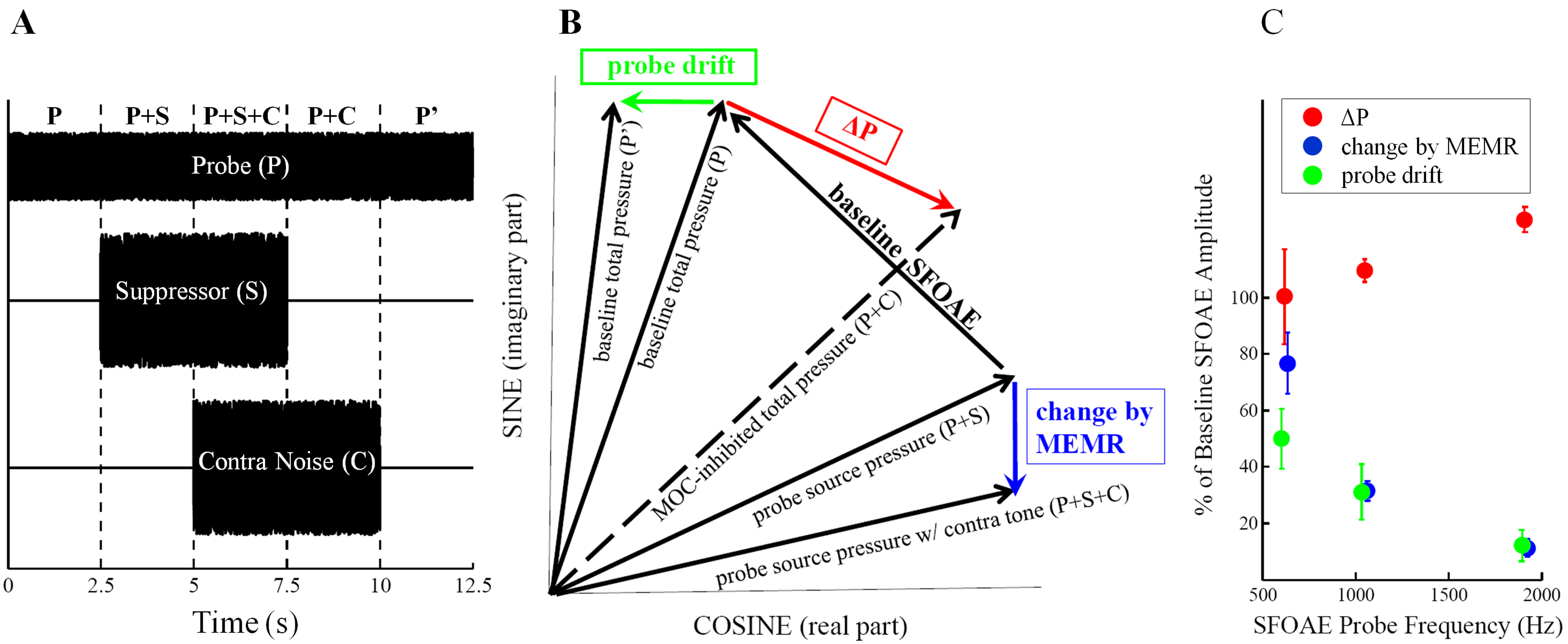

Supplement: Figure S2 — The suppression method for detecting MEM contraction. (A) Illustration of the experimental paradigm. A 40 dB SPL probe tone, a 65 dB SPL suppressor tone 0.1 octave below the probe tone, and a 68 dB SPL contralateral noise were presented for different durations in a 12.5-s window, segmenting it into five conditions: probe alone (P), probe plus suppressor (P+S), probe plus suppressor and contralateral noise (P+S+C), probe plus contralateral noise (P+C), and finally probe alone again (P′). Computing the vector difference between these conditions yields baseline SFOAE, pressure change induced by the middle-ear muscle reflex (MEMR) (blue arrow), contralateral noise-induced shift ΔP (red arrow) and probe drift (green arrow) (B). Exemplar results from subject WTPF42 are displayed (C). For two SFOAE probe frequencies (∼1000 and ∼2000 Hz), the magnitude of ΔP (red symbols) was substantially larger than that of, and therefore could not be explained by either MEMR-induced pressure change (blue symbols) or probe drift (green symbols). Hence ΔP was considered to be dominated by the MOC reflex. Error bars are one standard error. (TIF) [file pone.0018725.s002.tif]
